# Matching Meals to Body Clocks—Impact on Weight and Glucose Metabolism

**DOI:** 10.3390/nu9030222

**Published:** 2017-03-02

**Authors:** Amy T. Hutchison, Gary A. Wittert, Leonie K. Heilbronn

**Affiliations:** 1 Adelaide Medical School, The University of Adelaide, Adelaide SA 5000, Australia; amy.hutchison@adelaide.edu.au (A.T.H.); gary.wittert@adelaide.edu.au (G.A.W.); 2 South Australian Health and Medical Research Institute (SAHMRI), North Terrace, Adelaide SA 5005, Australia; 3 Robinson Research Institute, The University of Adelaide, North Adelaide SA 5006, Australia

**Keywords:** circadian rhythm, glucose metabolism, time-restricted feeding, type 2 diabetes, chronic disease risk

## Abstract

The prevalence of type 2 diabetes continues to rise worldwide and is reaching pandemic proportions. The notion that this is due to obesity, resulting from excessive energy consumption and reduced physical activity, is overly simplistic. Circadian de-synchrony, which occurs when physiological processes are at odds with timing imposed by internal clocks, also promotes obesity and impairs glucose tolerance in mouse models, and is a feature of modern human lifestyles. The purpose of this review is to highlight what is known about glucose metabolism in animal and human models of circadian de-synchrony and examine the evidence as to whether shifts in meal timing contribute to impairments in glucose metabolism, gut hormone secretion and the risk of type 2 diabetes. Lastly, we examine whether restricting food intake to discrete time periods, will prevent or reverse abnormalities in glucose metabolism with the view to improving metabolic health in shift workers and in those more generally at risk of chronic diseases such as type 2 diabetes and cardiovascular disease.

## 1. Overview

The rate of type 2 diabetes is rapidly increasing worldwide, and is predicted to be the main contributor to disease burden in Australia by 2023 [1]. Obesity, particularly abdominal obesity and fatty liver are major contributors to the development of type 2 diabetes. In addition to the effects of energy imbalance and suboptimal nutrient consumption, accumulating evidence suggests that circadian de-synchrony, defined as when physiological processes are out of alignment with internal clocks, may be a contributing, and modifiable, factor in the development of type 2 diabetes [2]. 

Almost all living organisms display circadian rhythms that cycle within a 24-h period. Disruption of circadian rhythms, either as a result of shifting the light/dark cycle, or from genetic manipulations (e.g., knockout of essential clock genes encoding circadian rhythmicity), result in metabolic disturbances that include weight gain, impaired glucose tolerance and reduced lifespan in mouse models [3,4]. In humans, shift workers are at increased risk of developing metabolic disorders, including obesity and type 2 diabetes [5,6,7,8]. However, associations do not show causation, and shift workers are also more likely to have a lower socio-economic status, smoke, consume more alcohol, and have a higher dietary fat intake [9]. In this review, we discuss the role of circadian de-synchrony, and interactions between this and the pattern of meal consumption, in weight gain, and glucose metabolism in mouse models and humans. We examine the evidence for whether small shifts in meal timing (such as skipping breakfast, or eating erratically, without consistent daily meal times) negatively impact glycaemic control and lipid metabolism. The current evidence for a benefit of time restricted feeding, a dietary pattern whereby food intake is confined to short windows of time, is also interrogated and implications for translation to practice presented.

## 2. Circadian Rhythms and Impacts on Metabolism

Circadian rhythms oscillate with near 24-h rhythmicity under constant conditions, and are entrainable by external cues. Central to the maintenance of the circadian clock is the suprachiasmatic nucleus (SCN) in the brain which is entrained by the light/dark cycle, via retinal photoreceptors in the retino-hypothalamic tract. The SCN is also entrained by the sleep/wake cycle, physical activity, and fasting and feeding periods [10,11,12]. Circadian clocks have now been identified in almost all tissues and cell types, where they regulate local metabolic processes, including glucose and lipid homeostasis, hormone release, immune response, gastrointestinal motility and digestive processes [13]. Whilst these “peripheral clocks” receive input from the SCN, their phase is sensitive to other external factors including nutrient availability [14]. 

The molecular basis of circadian timing is provided by transcriptional/translational feedback loops centred on the transcriptional activators; circadian locomotor output cycles kaput (CLOCK) and brain and muscle ARNT-like 1 (BMAL1), which act as positive elements in the feedback loop. CLOCK and BMAL1 drive transcription of six repressor encoding genes, three period genes (*per1, per2, per3*), two crypto-chrome genes (*cry1 and cry2*), the transcription factor *Rev-Erbα* and one promoter gene *RORα* [15]. This core molecular clock cycles with a near 24-h periodicity and more than 25% of the transcriptome, proteasome, and more recently the phospho-proteasome [16], has been shown to cycle in temporally orchestrated waves. Over the past decade, many studies have shown that whole-body, or tissue-specific, knockout of circadian clock genes will induce profound changes in metabolism. These findings include increased adiposity, impaired glucose tolerance and reduced lifespan [3,4]. Many of these gene knockouts also display aberrations in feeding patterns, and eat more during the day, which may independently contribute to changes in metabolism [3,4]. 

## 3. Metabolic Consequences of Circadian De-Synchrony (Humans)

Epidemiological studies show that shift workers are at increased risk of developing obesity and type 2 diabetes [17]. One prospective study in women showed that the increased risk of type 2 diabetes was only partly mediated by greater weight gain in shift workers [18]. Interestingly, the prevalence of metabolic syndrome was also higher in men who had previously engaged in shift work versus those who had never performed shift work [19]. This could indicate that these disturbances in metabolism are not entirely reversible. 

In humans, metabolic studies of simulated shift work show that shift work induces major disturbances in metabolism, independently of sleep restriction. Circadian misalignment that is induced by a 28 h “day”, increased blood glucose levels in healthy adults, even in the presence of increased insulin secretion. This protocol was sufficient to temporarily induce pre-diabetes in 3 out of 8 individuals [20]. Whilst this study shows that circadian misalignment impairs glucose control, a 28 h “day” cycle is not representative of typical shift-work. In another study, individuals were randomly assigned to a “circadian alignment” protocol, where they slept from 11 p.m. to 7 a.m. from day 1–7, or a “circadian misalignment” protocol, where they slept from 11pm to 7am from day 1–3, but were then shifted 12 h to sleep from 11 a.m. to 7 p.m. for days 4–8. In the circadian alignment condition, glucose tolerance declined over the day from breakfast (8 a.m.) to dinner (8 p.m.), congruent with typical circadian changes in insulin secretion and resistance, discussed later in this review. However, under the circadian misalignment protocol, glucose tolerance was lower, presumably as a result of lowered insulin sensitivity. Critically, prolonged exposure to circadian misalignment resulted in poorer glucose tolerance [21]. Both meals and sleep times were shifted, with a meal consumed at around midnight in the circadian misalignment condition. Despite attempts to match sleep between conditions, sleep duration and quality were reduced in the misalignment condition. The separate contributions of eating late at night, vs. exposure to light and not sleeping at night, have not yet been reported in humans. 

### Metabolic Consequences of Circadian De-Synchrony (Pre-Clinical Models) 

Feeding and fasting periods are important external synchronisers for peripheral oscillators. Studies in mice have shown that fasting for 24-h flattens more than 80% of rhythmically expressed transcripts in the liver [22]. One mechanism through which this occurs is by activation of 5′ AMP-activated protein kinase (AMPK), which phosphorylate*s cry*, targeting it for degradation. Fasting also inhibits mechanistic target of rapamycin (mTOR) activity, which impacts *per* stability [23], and induces circardian de-synchrony.

Studies have shown that feeding rodents solely during the day (when this nocturnal animal would normally sleep), increases body weight as compared to animals fed ad libitum [24]. Similar responses have been observed in mice that are fed a high fat diet, with significantly more weight gain and poorer glucose tolerance in mice that are fed during the day versus mice that are fed at night [25]. Interestingly, feeding mice three discrete “meals” in a configuration that mimics typical human meal times (the mouse equivalent of 7 a.m., 12 p.m. and 8 p.m., with slightly more calories provided at lunch and dinner than breakfast) led to a phase advancement of the peripheral clock [26]. The metabolic impacts of this were not assessed, but there was no effect on body weight. However, the short duration and the necessity of implementing a 20% caloric restriction overall to ensure timely meal completion, could have influenced this response.

Mice that are fed a high-fat diet (HFD), ad libitum*,* display dampened diurnal rhythms in food intake, and eat more during the resting phase [24]. This abnormal pattern of eating, and the lack of a defined fasting period disrupts the cyclic pattern of expression of peripheral clocks and downstream targets, and may explain at least some of the metabolic consequences that are observed as a result of a high fat diet [27,28]. Whilst discussion of the effects of nutrients on circadian rhythms is beyond the scope of this review, and this has been extensively reviewed by others [29], it is important to acknowledge that nutrients have the potential to act as zeitgebers. As such, the composition of the diet may impact the degree of de-synchrony that occurs. However, evidence in humans is scant, and requires further investigation. 

## 4. Glucose Metabolism, Gut Hormones and Circadian Rhythms

Like many other systems, postprandial glycaemia is under circadian regulation [30]. In humans, meal tests that are performed in the evening result in a hyperglycaemic response vs. identical meals that are given in the morning, even when there are identical fasting lengths between meals [31,32,33]. This impairment in glucose tolerance in the evening is the result of reduced insulin secretion, as well as peripheral and hepatic insulin resistance [32,34,35,36], which occurs independent of the sleep/wake and feeding/fasting cycles [36,37]. 

Anorexigenic hormones including glucagon-like peptide-1 (GLP-1), glucose-inhibitory peptide (GIP), peptide YY (PYY) and amylin, glucagon and insulin, and the orexigenic hormone ghrelin, also oscillate around anticipated meals. These gut hormones play a critical role in modulating gastric emptying, and the glycaemic response to meals. At night, gastric emptying slows [21]. Whether there are circadian rhythms in the release of gut hormones is still poorly described, although GLP-1 has received considerable interest. In a series of in vitro and in vivo experiments, a clear circadian pattern in the release of GLP-1 from rat and human intestinal L cells has been reported [38,39,40]. This pattern is altered by circadian disruptors, including constant light exposure, a Western diet and altered meal patterning (i.e., feeding during the day in rats). In rodents, clear diurnal GLP-1 rhythmicity is observed, in phase with insulin, with peak responses occurring when feeding is limited [39]. This response is entrained, since animals that are fed during the day (simulating a night-shift) showed a shift in GLP-1 peak, and a disturbed relationship between GLP-1, insulin and glucose concentrations [39]. In addition, exposure to constant light and feeding a high-fat, high-sucrose ‘Western’ diet, abrogated the normal rhythmic patterns of GLP-1 and insulin release and impaired glucose tolerance [38,39]. 

In humans, circadian-like patterns of GLP-1 levels, as well as a reduction in the amplitude of GLP-1 release in individuals with obesity and type 2 diabetes have been observed. However, these initial studies did not account for inter-meal intervals, or the caloric loads of the meals administered [41,42,43]. Subsequent studies that controlled these conditions, and fed participants identical mixed-nutrient test meals, 12 h apart, also showed rhythmic patterns in basal and post-prandial GLP-1 and insulin concentrations [40]. In contrast with rodents, GLP-1 and insulin responses did not change in parallel, with highest GLP-1 secretion observed at 2300 h whereas the highest insulin response was noted at 1100 h [40]. This study showed that exposure to 22 h of constant light dampened the patterns of GLP-1 and insulin release, in association with insulin resistance. Interestingly, these changes were not observed in participants maintained under the same sleep-deprivation protocol but within the normal light–dark period, which suggests a direct effect of light. Collectively, the data in both rodents and humans demonstrate a functional role of GLP-1 in the peripheral metabolic clock, and suggest that altered release of GLP-1, may be one mechanism that contributes to the metabolic perturbations that result from circadian de-synchrony. Further research is required to extend these observations in humans, and to establish the roles of irregular light-dark cycles on these pathways.

## 5. Can Smaller Misalignments in Meal Timing Impair Metabolic Processes?

As discussed, shift work, and eating significant amounts of food at night impairs glucose tolerance. However, many individuals do not entirely switch eating patterns from day to night. It is unclear whether smaller misalignments in meal timing, such as skipping breakfast, or consuming late night snacks, will induce similar impairments. It is also important to identify whether social jetlag, or eating and sleeping later on “weekends” is sufficient to impact metabolic health. Gill et al. [44] recently performed an observational study in 156 non-shift workers to examine typical human eating patterns. In this study, participants downloaded a smartphone application (app) and were asked to take a photo of each meal/beverage, just prior to consuming it, for 3 weeks. This time stamped when each food was eaten, and this information was uploaded to the investigators. There was a large variation in the number of meal events (4–15/day), and the average inter-meal interval was 3 h. More than half of the cohort reported eating over a 15 h time period each day (e.g., 0700–2200 h), with 75% of energy intake occurring in the afternoon and evening. This evidence is concerning given our knowledge that food intake entrains peripheral clocks, and that the eating during the day increases the risk of type 2 diabetes in mouse [27]. A recent study in humans examined the metabolic impacts of an identical 40% overfeeding diet as 3 meals per day or 3 meals and 3 snacks per day. Although, no differences in weight gain were observed, this study showed that increased meal frequency, in the presence of caloric excess, increased abdominal adipose tissue deposition, increased hepatic triglyceride content, reduced insulin-induced suppression of non-esterified fatty acid (NEFA) and reduced hepatic insulin sensitivity [45]. Of note, the snacks were consumed after each meal, and thus individuals in the snacking arm ate for longer each day, and later at night, which may have influenced this response. 

Given the known circadian oscillations in GLP1, insulin release and glycaemia, eating more food earlier in the day has been hypothesised to be optimal for overall glycaemic control in individuals with type 2 diabetes. A study in which a hypo-energetic diet was prescribed as a high energy breakfast (08:00 h, 3000 kJ), standard lunch (13:00 h, 2500 kJ) and low energy dinner (21:00 h, 900 kJ), or the reverse protocol, was prescribed to individuals with type 2 diabetes for one week each [46]. Postprandial lunchtime glycaemia was lowest and insulin and GLP-1 concentrations were highest, when participants had followed the high energy breakfast protocol. This suggests that eating more breakfast could produce optimal glucose control. However, this difference could also be the result of consuming more kilojoules at breakfast (i.e., a greater preload effect). In individuals with type 2 diabetes, skipping breakfast increases the peak glycaemic response to a subsequent lunch meal [47]. This is expected, given the well described literature of the second meal effect, i.e., the effect that the prior meal has on reducing the glycaemic response to the next meal [48]. However, skipping breakfast also increased the postprandial glycaemic response to a subsequent dinner meal, which was unexpected [47]. Skipping breakfast also reduced postprandial insulin and GLP-1 secretion and increased NEFA and glucagon concentrations at the subsequent dinner meal. This study was conducted over the course of a single day in individuals who regularly ate breakfast. There is some evidence to suggest that there is entrainment in the response to specific meal patterns over time [49].

## 6. Matching Food Intake with Body Clocks

Time restricted feeding (TRF) describes a dieting approach whereby food is available ad libitum for a short window of time each day. Mice that are fed a high-fat diet, under ad libitum conditions display dampened diurnal rhythms in food intake and resting metabolic rate. Conversely, providing mice with a high fat diet under time-restricted conditions (i.e., for 9–12 h), solely during the night, resets peripheral clocks, and abrogates many of the metabolic consequences of a HFD. This includes restoring diurnal oscillations in resting energy expenditure and hepatic glucose metabolism [27]. A similar response has been observed in diet-induced obese mice, with TRF reducing hyperinsulinemia, hepatic steatosis, and inflammation [50]. Interestingly, when lean animals were switched to a TRF-HFD but allowed ad libitum access to a high fat diet for 2 consecutive days per week, (simulating a “weekend”), lean body weights and metabolic profiles were maintained [50]. A number of TRF studies have shown positive effects in various rodent models of metabolic disease, with the most commonly selected time being 8 h of food access, during the active phase [28,51]. These studies suggest that TRF will negate the metabolic consequences of poor dietary habits, at least in mouse. 

The impacts of TRF in humans are less clear, and prospective randomised controlled trials testing this concept are limited. From the epidemiological data, individuals who report consuming more than one third of daily energy intake at the evening meal have double the risk of obesity as compared to individuals who report consuming more than a third of energy intake by 1200 h [52]. Eating lunch after 1500 h was also predictive of poorer weight loss and changes in markers of insulin sensitivity during a 20-week dietary intervention, independently of self-reported 24-h caloric intake [53]. In a randomised trial, participants assigned to consume more of their allotted kilojoules at breakfast lost more weight compared with those who consumed the majority of kilojoules at dinner [54]. Similarly, a hypoenergetic diet consumed as breakfast and lunch produced greater reductions in weight, hepatic lipid content, and greater improvements in glucose tolerance versus 6 meals/day in individuals with type 2 diabetes after 12 weeks [55]. Of note, those in the 2 meal per day condition would have fasted for longer prior to metabolic testing, which may have compounded the observed differences. Together, with the evidence presented above, it appears that consuming more energy in the morning, as opposed to later in the day, is beneficial for glycaemic control. However, it is unclear whether this is causal in the development of type 2 diabetes, and longer term randomised controlled trials comparing the metabolic impacts of this are necessary to definitively answer this question.

There are many observational studies of individuals who undertake the Islamic ritual of fasting during the month of Ramadan [56,57,58,59,60,61,62]. Ramadan is essentially a time restricted feeding protocol that requires individuals to abstain from eating and drinking during daylight hours. Given the animal data presented, the switch to a predominately night time pattern of food consumption that characterises Ramadan, could be predicted to adversely impact metabolic health. Conversely, most studies report favourable improvements in blood lipids, including reductions in total and low density lipoprotein (LDL)-cholesterol, triglycerides, and increases in high density lipoprotein (HDL)-cholesterol [56,57,60,61,62]. Some of these health benefits may be due a mild energy restriction, and modest weight loss that is typically observed in response to Ramadan fasting [56,57,61,62]. However, postprandial hyperglycaemia [63], increased fasting blood glucose and deterioration in glycaemic control [57,64] have also been reported. We speculate that implementing a TRF protocol at night will be beneficial for regulation of body weight, and cardiovascular outcomes, but not for glycaemic control. 

One controlled study has examined the effects of implementing an evening TRF protocol on metabolic health outcomes. In this study, lean individuals were asked to limit all food intakes to a 4-h window early in the evening (1700–2100 h), vs. eating the same diet as breakfast, lunch and dinner for 8-weeks each [65,66]. All food intakes were monitored within a metabolic kitchen. Despite eating identical foods, the TRF condition resulted in small but significant reductions in body weight and fat mass, and improved cardiovascular profiles, including increased HDL and reduced triacylglycerol [65]. These changes were independent of diet composition, since dietary cholesterol and fatty acids were carefully matched in each dietary condition. In spite of modest weight loss in the TRF condition, fasting blood glucose levels were increased, and impaired glucose tolerance [66]. There were no differences in insulin response. This study demonstrates that limiting energy intake to late in the day is detrimental for glucose control in humans. This outcome may have been influenced by the greater number of kilojoules that were consumed closer to the time of testing in one condition (i.e., 100% calories between 1700 and 2100, vs. 30%–40% in the 3 meals/day condition). Alternatively, the glucose test was performed at a time that participants were no longer accustomed to eating, which could have impacted results [49]. In a similarly styled study, healthy, lean men underwent an alternate day fasting protocol, fasting from 2200 h until 1800 h the following day. This 20 h/day fast meant that re-feeding occurred later in the day. Despite this pattern of meal intake, insulin sensitivity was increased, although no changes in body weight, fasting blood glucose or insulin were noted [67]. The reason for the disparate results are unclear, but may be related to participant characteristics or the amount of energy consumed in the evening.

Two other TRF protocols have also been piloted to date. In one, 8 individuals who were obese and reported eating for at least 14 h/day were recruited [44]. Individuals were instructed to limit food intakes to 10–11 h/day, with no other dietary instruction. TRF resulted in 3.3 kg weight loss after 16 weeks, which was maintained for 12 months. Of note, the precise TRF schedule was self-selected, and participants shortened both ends of their day (avg. 1000–2030 h), and there was no control group. In another study, lean healthy men ate ad libitum vs. 13 h/day TRF (0600–1900) for 2 weeks each. The TRF study condition resulted in less food consumption, and a −0.4 kg weight loss, compared with a gain of +0.6 kg under ad libitum conditions [68]. Metabolic health outcomes were not reported in either publication, which makes it difficult to establish whether beneficial health effects, beyond weight loss, exist. The limited number of studies, with small sample sizes, lack of adequate controls, as well as the lack of data reporting the effects of TRF in individuals who are obese highlights the necessity of further research in this area. 

A final aspect that remains is whether eating erratically will alter glucose control. To our knowledge two studies in humans have partially investigated this concept. Participants were asked to eat between 3–9 meals per day, or eat 6 meals/day at the same time each day for 2 weeks each. The irregular meal pattern caused insulin resistance in response to a high-carbohydrate breakfast meal in women who are lean [69] and obese [70], although fasting blood glucose was not different between meal conditions. These studies suggest that erratic eating patterns may also induce insulin resistance, but longer term studies are required. 

## 7. Conclusions 

There is a general belief that consumption of more energy throughout the day is preferable to evening consumption. Few studies have examined this prospectively in humans, or for any length of time. Nonetheless, time restricted feeding has shown promise as a tool to mitigate the metabolic sequelae of diet induced obesity in mouse models. Good quality evidence for TRF as a dietary approach to improve glucose control in humans is lacking. Controlled trials are necessary, and must determine if there is adaptation in the approach, whilst keeping in mind the practicality of translating this approach into the community. 
